# For Trigeminal Neuralgia

**Published:** 1890-11

**Authors:** 


### For Trigeminal Neuralgia.

Dr. E. C. Seguin in a lecture to the Medical Society of the University of Toronto, recommends for trigeminal neuralgia Duquesnel's crystallized aconitine. A pill of 1-200 of a grain is given twice a day to women, three times a day to men, gradually increased until two pills every three hours are given and physiological effects are obtained. These are tingling and numbness in the face, tongue, and extremities, with a sense of chilliness, usually marked along the spine. Having found the dose of toleration, it should be kept up daily for several weeks after the pain has ceased. With this remedy, red iodide of mercury should be given, dose gradually increased from 1–20 to 1–5 or 1–6 of a grain combined with iodide of potassium. As a case of this affection approaches cure there are sometimes spots of exquisite sensitiveness on the face and head, which, if irritated will produce a return of the neuralgia. Blistering these spots will cause them to disappear. Cod liver oil should also be given in this disease.

